# DNA methylation analysis in plasma for early diagnosis in lung adenocarcinoma

**DOI:** 10.1097/MD.0000000000038867

**Published:** 2024-07-12

**Authors:** Yulin Jin, Rongguo Lu, Feng Liu, Guanyu Jiang, Ruixin Wang, Mingfeng Zheng

**Affiliations:** a Department of Thoracic Surgery, Wuxi People’s Hospital Affiliated to Nanjing Medical University, Wuxi, Jiangsu, China.

**Keywords:** DNA methylation, lung adenocarcinoma, plasma, RASSF1A, SHOX2

## Abstract

**Background:**

Lung adenocarcinoma (LUAD) represents the most prevalent type of lung cancer. SHOX2 and RASSF1A methylation have been identified as important biomarkers for diagnosis and prognosis of lung cancer. Bronchoalveolar lavage fluid (BALF) exhibits good specificity and sensitivity in diagnosing pulmonary diseases, but its acquisition is challenging and may cause discomfort to patients. In clinical, plasma samples are more convenient to obtain than BALF; however, there is little research on the concurrent detection of SHOX2 and RASSF1A methylation in plasma. This study aims to assess the diagnostic value of a combined promoter methylation assay for SHOX2 and RASSF1A in early-stage LUAD using plasma samples.

**Methods:**

BALF and blood samples were obtained from 36 early-stage LUAD patients, with a control group of nineteen non-tumor individuals. The promoter methylation levels of SHOX2 and RASSF1A in all subjects were assessed using the human SHOX2 and RASSF1A gene methylation kit.

**Results:**

The methylation detection rate of SHOX2 and RASSF1A in plasma was 61.11%, slightly lower than that in BALF (66.7%). The Chi-square test revealed no significant difference in the methylation rate between BALF and plasma (*P* > 0.05). The area under the receiver operating characteristic (ROC) curve analysis for blood was 0.806 (95% CI, 0.677 to 0.900), while for BALF it was 0.781 (95% CI, 0.649 to 0.881). Additionally, we conducted an analysis on the correlation between SHOX2 and RASSF1A methylation levels in plasma with gender, age, tumor differentiation, pathologic classification, and other clinicopathological variables; however, no significant correlations were observed.

**Conclusions:**

Measurement of SHOX2 and RASSF1A methylation for early diagnosis of LUAD can be achieved with high sensitivity and specificity by using plasma as a substitute for BALF samples.

## 1. Introduction

According to the cancer statistics released by the National Cancer Center of China in 2022, lung cancer exhibits the highest incidence and mortality rates, with lung adenocarcinoma (LUAD) being its most prevalent subtype.^[1]^ The 5-year survival rate for lung cancer is merely 15% to 19% across all stages, primarily due to the inconspicuous clinical manifestations and pathological characteristics of this disease, and there are few effective and inexpensive methods for early detection and screening.^[2]^ However, patients who undergo surgical resection for early-stage LUAD demonstrate a favorable prognosis, particularly at stage I, where the 5-year survival rate can be elevated to 81% to 85%.^[3,4]^ Although low-dose computed tomography has gained widespread acceptance as a reliable screening tool for lung cancer, its high false-positive rates and subsequent overdiagnosis limit its clinical application.^[5–7]^ Therefore, it is crucial to explore novel noninvasive approaches that can enhance the detection rate of early-stage LUAD.

The analysis of molecular changes in body fluids associated with lung cancer has been demonstrated as a safe and economical approach for the detection of lung cancer.^[8]^ Recent studies have revealed that gene methylation is closely linked to the occurrence and progression of malignant tumors, thus holding potential for diagnosis and prognosis.^[9,10]^ DNA methylation, a major epigenetic mechanism regulating gene expression and cellular characteristics, exhibits relative stability and can be detected not only in tissues but also in blood, urine, saliva, and other bodily fluids.^[11]^ With the development of methylation-specific PCR and other technologies, the methylation of many genes has been proved to be a promising molecular marker for early diagnosis of cancers.^[12–14]^ Currently, promoter methylation of short-stature homeobox gene II (SHOX2) and RAS association domain family 1A subtype (RASSF1A) has been identified as diagnostic and prognostic biomarkers in lung cancer.^[2,15,16]^

Bronchoalveolar lavage fluid (BALF), being a highly specific and sensitive sample for the diagnosis of pulmonary diseases, has been extensively utilized in the early detection of lung cancer. Lou et al showed that SHOX2 and RASSF1A gene methylation in BALF were significantly different between the cancer group (n = 284) and control group (n = 38).^[17]^ Other studies have also confirmed that combined detection of these 2 genes can assist in diagnosing lung cancer, with sensitivities ranging from 71.5% to 83.2% and a specificity of 90% to 100%.^[18,19]^ However, acquiring BALF is clinically uncomfortable for patients and may lead to complications such as bleeding, laryngeal edema or hoarseness, and asthma attacks. In contrast, blood samples are easier to obtain, and individual gene methylation of SHOX2 or RASSF1A has been analyzed in blood samples.^[20,21]^ Nevertheless, few studies have focused on the combined detection of plasma SHOX2 and RASSF1A methylation. Therefore, this study aims to investigate the value and significance of combining plasma SHOX2 and RASSF1A methylation in aiding the diagnosis of LUAD.

## 2. Methods

### 2.1. Study subjects and samples

A total of 36 patients with LUAD and 19 non-tumor patients were enrolled in this study. Informed consent was obtained from all participants, and the use of patient samples in this study was approved by the Ethics Committee of Wuxi People Hospital Affiliated to Nanjing Medical University (No.KY21084). The subjects were treated in accordance with the Declaration of Helsinki.

Blood samples were collected from patients using vacuum collection vessels (CYTOMARK, UK), which stored at 4°C and processed within 48 hours. Plasma separation was performed by centrifuging blood samples at 1600 g for 10 minutes. BALF was obtained by flushing the affected lung segment with normal saline (20–40 mL) during fiberoptic bronchoscopy. Clinical parameters such as gender, age, differentiation degree, and stage were extracted from medical records and are presented in Tables 1 and 2.

**Table 1:** Clinicopathological characteristics of 36 patients with early-stage LUAD.

Characteristics	N (%)
Sex	
Male	11 (30.6)
Female	25 (69.4)
Age	
<60	21 (58.3)
≥60	15 (41.7)
Pathologic classification	
Microinvasive adenocarcinoma	22 (61.1)
Invasive adenocarcinoma	8 (22.2)
Other adenocarcinoma	6 (16.7)
Differentiation degree	
Poorly/Moderately	8 (22.2)
Well	28 (77.8)
T Stage	
Tis	4 (11.1)
T1mi/T1a	23 (62.84)
T1b	6 (16.7)
T1c	3 (8.3)

LUAD = lung adenocarcinoma, N = total number of patients, T = tumor size.

**Table 2:** Clinicopathological characteristics of 19 non-tumor controls.

Characteristics	N (%)
Sex	
Male	9
Female	10
Age	
<60	12
≥60	7

Non-tumor controls included patients with pulmonary bulla and pneumonia.

### 2.2. DNA extraction and bisulfite treatment

The DNA extraction was performed using the TIANamp Genomic DNA Kit (Tiangen Biotech Co., Ltd., Beijing China) on 10 mL of BALF that had been centrifuged at 8000 rpm for 10 minutes. For plasma samples, cell-free DNA (cfDNA) was extracted following the manufacturer instructions with the MagicPure Cell-Free DNA Kit II (TransGen Biotech., China). Subsequently, unmethylated cytosines were converted to uracil through sodium bisulfite modification using the EZ DNA Methylation-direct kit (Zymo Research). The resulting DNA samples were either immediately analyzed or stored at −80°C.

### 2.3. Methylation detection

The commercial SHOX2 and RASSF1A Methylation Detection Kit (Tellgen Co., Shanghai, China) was employed for the detection of methylation status in all samples. Methylated SHOX2 and RASSF1A DNA plasmids were utilized as internal controls. Quantitative real-time PCR (qPCR) was performed using the ABI 7500 Real-time PCR System, and result interpretation followed the manufacturer instructions. An amplification curve of the FAM fluorescence signal with a smooth “S” shape and a threshold cycle (CT) < 35 indicated a positive result for RASSF1A methylation [R+], CT ≥ 35 indicated a negative result for RASSF1A methylation [R-]. Similarly, a positive result for SHOX2 methylation [S+] was determined by an amplification curve of the VIC fluorescence signal with a smooth “S” shape and CT < 32, whereas a negative result [S−] had CT ≥ 32. When neither gene exhibited methylation, it was classified as negative; otherwise, it was considered positive.

### 2.4. Statistical analysis

Statistical analyses were conducted using SPSS 27.0 software package (SPSS Inc., Chicago, IL). The detection rates of the aberrant methylation of the SHOX2 and RASSF1A panel between BALF and plasma were analyzed using Fisher or χ^2^ test. MedCalc software was utilized for receiver operating characteristic (ROC) curve analysis, with calculation of the area under the ROC (AUC) to determine the potential of plasma DNA methylation. *P* values below .05 were considered statistically significant.

## 3. Results

### 3.1. The characteristics of the LUAD patients

A total of 36 early-stage LUAD patients were enrolled in this study, comprising 11 (30.6%) males and 25 (69.4%) females (shown in Table 1). Among them, 21 (58.3%) patients were younger than the median age of 60, while the remaining 15 (41.7%) patients were older than or equal to this age threshold (range: 31–76). Based on pathological classification, microinvasive adenocarcinomas accounted for 22 (61.1%) cases, invasive adenocarcinomas for 8 (22.2%), and other types of adenocarcinomas for the remaining 6 cases (16.7%). Histopathologically grading the primary tumors revealed that among these patients, there were poorly/moderately-differentiated tumors in a group of 8 individuals (22.2%), whereas well-differentiated tumors constituted a majority with twenty-eight cases (77.8%). Tumor staging showed that 4 tumors were classified as Tis stage (11.1%), twenty-three as T1mi/Tla stage (62.84%), 6 as T1b stage (16.7%), and 3 as Tic stage (8.3%).

Additionally, a control group consisting of nineteen individuals without evidence of LUAD was included in this study; it comprised 7 pulmonary bulla patients and twelve pneumonia patients (Table 2). In terms of gender distribution within the control group, 9 participants were male while twelve participants were under sixty years old.

### 3.2. Correlation of SHOX2 and RASSF1A methylation with LUAD BALF and plasma

**Figure 1.**
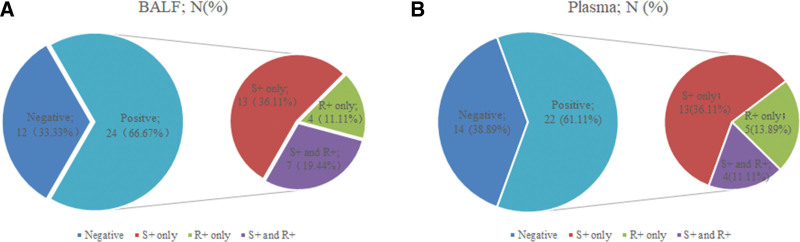
Results of the individual SHOX2/RASSF1A and combined methylation detection in 36 LUAD patients. (A) Positive rate of DNA methylation in BLAF samples. (B) Positive rate of DNA methylation in plasma samples. BALF = Bronchoalveolar lavage fluid, LUAD = lung adenocarcinoma, RASSF1A = RAS association domain family 1A subtype, SHOX2 = short stature homeobox gene II.

The combined methylation assay of SHOX2 and RASSF1A was performed on BALF and plasma samples obtained from 36 patients with early-stage LUAD. The positive cases for individual assays of SHOX2, RASSF1A, and the combined methylation were depicted in Figure 1. In the tumor tissues analyzed (n = 36), the occurrence of SHOX2 and RASSF1A methylation is 66.7%. The occurrence rates of individual SHOX2, individual RASSF1A, and combined methylation in BALF samples were found to be 36.11%, 11.11%, and 19.44%, respectively. Similarly, in plasma samples (n = 36), the occurrence of SHOX2 and RASSF1A methylation is 61.11%, slightly lower than those observed in BALF samples. The occurrence in plasma samples of the individual SHOX2, individual RASSF1A and combined methylation was 36.11%, 13.89% and 11.11%, respectively.

The χ^2^ test results indicated that there was no significant difference between the detection rates of methylation in BALF versus plasma (*P* = .6175). Furthermore, no positive results for SHOX2 or RASSF1A methylation were detected in plasma samples from 19 non-tumor patients.

### 3.3. ROC curve analysis

**Figure 2.**
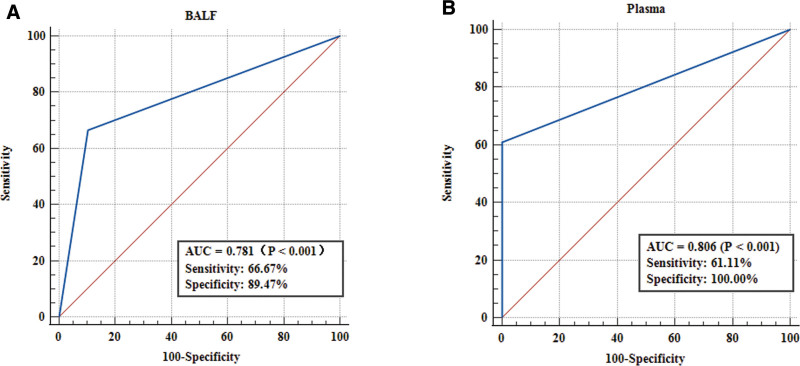
ROC curve analysis of DNA methylation in 36 early LUAD and 19 non-tumor patients. The area under the ROC curve (AUC) conveys its accuracy for diagnosing LUAD. (A) The ROC curve of DNA methylation in BLAF samples. (B) The ROC curve of DNA methylation in plasma samples. BALF = Bronchoalveolar lavage fluid, LUAD = lung adenocarcinoma, ROC = receiver operating characteristic curve.

The primary criterion used to evaluate the diagnostic value was the area under the receiver operating curve (ROC) analysis, specifically referred to as the area under the curve (AUC). Therefore, it was employed for comparing the diagnostic efficacy of SHOX2/RASSF1A methylation in both BALF and plasma samples (Fig. 2). The AUC values obtained from ROC analysis were 0.781 (95% CI, 0.649–0.881) and 0.806 (95% CI, 0.677–0.900), respectively.

### 3.4. Association of SHOX2 and RASSF1A methylation in BALF and plasma with clinicopathological variables in early LUAD

The relationship between SHOX2/RASSF1A methylation and clinicopathological variables in BALF and plasma of 36 patients with early LUAD was assessed using Fisher or χ^2^ test (Table 3). Results showed that SHOX2/RASSF1A methylation in either BALF or plasma had no statistically significant correlation with any of the collected clinicopathological variables, including gender, age, tumor pathological type, and tumor differentiation degree (*P* > .05). These findings further support the important value of plasma SHOX2 and RASSF1A methylation in the diagnosis of LUAD.

**Table 3:** The association between SHOX2/RASSF1A DNA methylation detected in early LUAD patients and clinicopathological variables was assessed using Fisher or χ^2^ test.

Characteristics	SHOX2/RASSF1A	P/N
BALF	Plasma
Sex		
Male	9/2	9/2
Female	15/10	13/12
*P*	.2072	.0955
Age*		
<60	13/8	11/10
≥60	11/4	11/4
*P*	.4795	.2100
Pathologic classification		
Microinvasive adenocarcinoma	14/8	11/11
Invasive adenocarcinoma	8/2	8/2
Other adenocarcinoma	2/2	3/1
*P*	.4988	.2266
Differentiation degree		
Poorly/Moderately	7/1	7/1
Well	17/11	15/13
*P*	.1623	.0869

LUAD = lung adenocarcinoma, RASSF1A = RAS association domain family 1A subtype, SHOX2 = short stature homeobox gene II.

## 4. Discussion

Numerous studies have demonstrated the association between aberrant methylation changes of the SHOX2/RASSFIA genes and cancer diagnosis.^[22,23]^ The Methylated Human SHOX2 and RASSF1A Gene Detection Kit (Tellgen Co. Ltd., Shanghai, China) has been utilized for early lung cancer diagnosis and differentiation of benign and malignant pulmonary nodules.^[24,25]^ Moreover, this kit can be employed to detect a variety of sample types, including BALF, tissue, pleural fluid, sputum, etc. In this study, we collected BALF samples from 36 patients with early LUAD and assessed their methylation status. The results showed that the DNA methylation sensitivity in BALF was 66.7%, slightly lower than the manufacturer-provided range of 71.5–83.2%. This discrepancy may be attributed to either the small sample size or by the single type of lung cancer analyzed in our study. And studies have shown that SHOX2/RASSF1A methylation exhibits higher sensitivity to small cell lung cancer (SCLC) and squamous cell carcinoma (SCC) than adenocarcinoma.^[26]^

Although BALF has been extensively utilized for early lung cancer diagnosis, the procedure to obtain BALF remains uncomfortable for patients. Some studies have found the 2 samples, peripheral blood and BALF, have similar sensitivity and specificity in distinguishing benign lesions from lung cancer.^[27]^ Hence, we aimed to investigate the methylation detection in blood samples as an early noninvasive biomarker for LUAD. Previous studies have indicated suboptimal sensitivity in detecting individual SHOX2 or RASSF1A methylation in plasma.^[20,23]^ Therefore, we combined the methylation detection of both SHOX2 and RASSF1A in plasma and compared it with that of BALF. The methylation sensitivity observed in plasma (61.11%) was slightly lower than that in BALF (66.7%), but this difference did not reach statistical significance (*P* > .05). The ROC analysis demonstrated that SHOX2 and RASSF1A methylation in plasma could serve as a promising noninvasive auxiliary diagnostic biomarker for early-stage LUAD (AUC = 0.806), and the diagnostic efficiency was better than BLAF (AUC = 0.781). Furthermore, we examined the correlation between SHOX2/RASSF1A methylation levels in plasma and clinicopathological variables such as gender, age, tumor pathologic classification, and degree of tumor differentiation; however, no significant associations were found. These results are consistent with other studies.^[26]^

There are certain limitations in our research. First, the inclusion of only 36 early LUAD patients may restrict the generalizability of our findings. Additionally, it would be preferable to have a control group consisting of healthy individuals to accurately assess specificity and sensitivity; however, obtaining BALF samples from normal subjects poses challenges. Moreover, to obtain a smooth PCR curve, it may be necessary to increase the volume of the plasma sample to extract enough cell-free DNA for accurate detection. Future studies could focus on exploring alternative methylation kits for SHOX2 and RASSFIA genes in plasma while also expanding the sample size by including more LUAD patients for enhanced accuracy.

## 5. Conclusion

Measurement of SHOX2 and RASSF1A methylation for early diagnosis of LUAD can be achieved with high sensitivity and specificity by using plasma as a substitute for BALF samples.

## Author contributions

**Conceptualization:** Yulin Jin, Rongguo Lu, Mingfeng Zheng.

**Data curation:** Yulin Jin, Rongguo Lu, Feng Liu, Guanyu Jiang.

**Formal analysis:** Feng Liu, Guanyu Jiang.

**Funding acquisition:** Yulin Jin.

**Investigation:** Feng Liu.

**Methodology:** Rongguo Lu, Feng Liu, Guanyu Jiang, Ruixin Wang, Mingfeng Zheng.

**Resources:** Ruixin Wang.

**Validation:** Guanyu Jiang.

**Writing – original draft:** Yulin Jin.

**Writing – review & editing:** Mingfeng Zheng.
